# 

**Published:** 1917-12

**Authors:** 


### INDEX.

Appendicitis in its relation to dyspepsia, Chronic—H. Goodwyn, 18.

**Books, Reviews of—**

Appendicitis, Acute—C. H. Whiteford, 135.
Bristol Royal Infirmary, A History of the —G. M. Smith, 129.
Bulkley, L. D.—Cancer, its cause and treatment, vol. i., 101.
Bulkley, L. D.—Cancer, its cause and treatment, vol. ii., 135.
Cancer, its cause and treatment, vol. i.—L. D. Bulkley, 101.
Cancer, its cause and treatment, vol. ii.—L. D. Bulkley, 135.
Cancer, The mortality from, throughout the world—F. L. Hoffman, 104.
Childhood, Common disorders and diseases of—G. F. Still, 23.
Cleft palate and hare lip—Sir W. A. Lane, 105.
Clinical Methods—R. Hutchinson and H. Rainy, 22.
Dawson, E. R.—Causation of sex in man, 134.
Gee, S.—Medical lectures and clinical aphorisms, 96.
Hartenberg, P.—Treatment of neurasthenia, translated by E. Playfair, 99.
Health, The nation's. The stamping out of venereal disease—Sir M. Morris, 133.
History of the Bristol Royal Infirmary, A. —G. M. Smith, 129.
Hurst, A. F.—Medical diseases of the war, 97.
Hutchinson, R., and H. Rainy—Clinical methods, 22.
Index-Catalogue of the library of the Surgeon-General's Office, United States army, vol. 21, 105.
Infectious diseases in schools, A code of rules for the prevention of, 105.
Kettle, E. H.—The pathology of tumours, 102.
Kidd, F.—Common diseases of the male urethra, 133.
Lane, Sir W. A.—Cleft palate and hare lip, 105.
Laurie, Jessie M.—War cookery book for the sick and wounded, 106.
Lead poisoning, from the industrial, medical, and social points of view—Sir T. Oliver, 98.
Lejars, F.—Urgent surgery, translated by D. S. Dickie, 23.
Medical annual, The, 1917—133.
Medical diseases of the war—A. F. Hurst, 97.
Medical lectures and clinical aphorisms—S. Gee, 96.

**Books, Reviews of** (*continued*)—

Military surgery—D. P. Penhallow, 100.
Morison, R.—Surgical contributions, vol. i. and ii., 104.
Morris, Sir M.—The nation's health. The stamping out of venereal disease, 133.
Mortality from cancer throughout the world—F. L. Hoffman, 104.
Neckwear, Dangers in—W. G. Walford, 132.
Nervous system, Diseases of the—C. Thomson, 97.
Neurasthenia, Treatment of—Dr. P. Hartenberg, translated by E. Playfair, 99.
Oliver, Sir T.—Lead poisoning from the industrial, medical, and social points of view, 98.
Pathology of tumours, The—E. H. Kettle, 102.
Penhallow, D. P.—Military surgery, 100.
Pneumonia, Traumatic, and traumatic tuberculosis—F. P. Weber, 22.
Riviere, C.—The pneumothorax treatment of pulmonary tuberculosis, 134.
Sex in man, Causation of—E. R. Dawson, 134.
Smith, G. M.—A history of the Bristol Royal Infirmary, 129.
Still, G. F.—Common disorders and diseases of childhood, 23.
Surgery, Manual of, vol. i. and ii.—A. Thomson and A. Miles, 104.
Surgery, Military — D. P. Penhallow, 100.
Surgery, Urgent, vol. ii.—F. Lejars, translated by D. S. Dickie, 23.
Surgical contributions, vol. i. and ii.—R. Morison, 104.
Sutherland, H. G.—Pulmonary tuberculosis in general practice, 22.
Thomson, A. and A. Miles—Manual of surgery, vol i. and ii., 104.
Thomson, C.—Diseases of the nervous system, 97.
Tuberculosis in general practice, Pulmonary —H. G. Sutherland, 22.
Tuberculosis, The pneumothorax treatment of pulmonary—C. Riviere, 134.
Tumours, The pathology of—E. H. Kettle, 102.
Urethra, Common diseases of the male—F. Kidd, 133.
Walford, W. G.—Dangers in neckwear, 132.
War cookery book for the sick and wounded, A.—Jessie M. Laurie, 106.
Weber, F. P.—Traumatic pneumonia and traumatic tuberculosis, 22.
Whiteford, C. H. — Acute appendicitis, 135.

Brew, R. H.—Obituary notice of, 110.
Bullen, F. St. John—Obituary notice of, 46.
Bush, Lieut.-Col. P. — Note on, 109.

Carrel-Dakin method, The prevention of sepsis in war wounds, with special reference to the—Dr. J. Swain, 68.
Clarke, Dr. J. M.—Gunshot wounds of the peripheral nerves, 57.
Connellan, P. S.—War surgery in an Indian general hospital in Mesopotamia, 113.

Davies, Dr. D. S.—Notes on plague in Bristol, 1916, 16.
Drugs and preparations for the sick, New, 32.
Dyspepsia, Chronic Appendicitis in its relation to dyspepsia—H. Goodwyn, 18.

Editorial notes, 24, 107.

Fatigue induced by labour—A. F. S. Kent, 47.
Fisher, Capt. A. G. T., Military honour conferred on, 107.

Glycerine, Restrictions on the use of, medicinal, 30.
Goodwyn, H.—Chronic appendicitis in its relation to dyspepsia, 18.
Gunshot wounds of the peripheral nerves—Dr. J. M. Clarke, 57.

Health, Ministry of (Editorial notes), 28.
Hill, W. J., Obituary notice of, 136.
Honours, War (Editorial notes), 27.

Kent, A. F. S.—Fatigue induced by labour, 47.
Keogh, Sir Alfred, Honour conferred on, 29.

Labour, Fatigue induced by—A. F. S. Kent, 47.
Lansdown, F. Poole, Obituary notice of, 42.

Mesopotamia, War surgery in an Indian general hospital in—P. S. Connellan, 113.
Morton, Dr. C. A.—Acute pancreatitis, with special reference to its treatment, and a record of three cases presenting unusual features, 80.

New drugs and preparations for the sick, 32.
Nixon, Capt. J. A., Military honour for, 109.

Obituary notices, 35, 110, 136.
Openshaw, E. H., Obituary notice of, 110.

Pancreatitis, Acute, with special reference to its treatment, and a record of three cases presenting unusual features—Dr. C. A. Morton, 80.
Pelvic inflammation in women, Rectal diverticula as a causative factor in—Dr. W. C. Swayne, 91.
Peripheral nerves, Gunshot wounds of the—Dr. J. M. Clarke, 57.
Pinniger, W. J. H., Obituary notice of, 136.
Plague in Bristol, 1916, Notes on—Dr. D. S. Davies, 16.
Plague outbreak "Nipped in the bud," 19.
Presidents, Death of three past (Editorial notes), 24.
Prowse, Lieut.-Col. A. B., Note on, 109.

Rectal diverticula as a causative factor in pelvic inflammation in women—Dr. W. C. Swayne, 91.
Roxburgh, R., Obituary notice of, 35.

Short, Dr. L. J., Introduction to a discussion on the diagnosis of tuberculosis, 1.
Smith, G. Munro, Obituary notice of, 36.
Students, Medical, and the war, 30.
Surgery, War, in an Indian general hospital in Mesopotamia—P. S. Connellan, 113.
Swain, Dr. J.—The prevention of sepsis in war wounds, with special reference to the Carrel-Dakin method, 68.
Swayne, Dr. W. C.—Rectal diverticula as a causative factor in pelvic inflammation in women, 91.
Swayne, Prof. W. C., Note on, 107.

Tuberculosis, Introduction to a discussion on the diagnosis of—Dr. L. J. Short, 1.

War Surgery in an Indian general hospital in Mesopotamia—P. S. Connellan, 113.
Wounds, with special reference to the Carrel-Dakin method, The prevention of sepsis in war—Dr. J. Swain, 68.

